# DTC-and-Me: Patient, Provider, Proteins and Regulators

**DOI:** 10.3390/jpm4010079

**Published:** 2014-03-18

**Authors:** Fintan R. Steele, Larry Gold

**Affiliations:** SomaLogic Inc., 2945 Wilderness Pl., Boulder, CO 80301, USA; E-Mail: lgold@somalogic.com

**Keywords:** proteomics, personalized medicine, SOMAmer, aptamer, SOMAscan, diagnostics, direct-to-consumer

## Abstract

The yet-unrealized potential for more “personalized” Direct-to-Consumer (DTC) tests to fundamentally alter the practice and economics of healthcare is undeniable. However, there are also many challenges to be met, including the herculean task of ensuring that the information provided by such tests is scientifically sound and, ideally, medically actionable. We consider recent events in DTC testing and suggest a “thought experiment” of an approach that could ultimately meet the needs of patients, providers and regulatory authorities.

## 1. Introduction

The development and marketing of Direct-to-Consumer (DTC) diagnostic or “health information” tests are redefining the boundaries of the medical and consumer cultures, in ways that have only begun to be recognized [1,2,3]. Both the general and the scientific/medical media are filled with a range of narratives about the value of this phenomenon, from the belief that medicine is finally being fundamentally transformed into something truly personalized and “patient-empowered” [4], to beliefs that a veritable Pandora’s Box has been opened, unleashing a host of unintended consequences [5]. Add to this range of beliefs the relatively complex science that underlies these emerging products, and it is not surprising that so many different strong opinions vie for the attention of the consumer who is the ultimate target, befuddle the healthcare provider challenged to incorporate this information into practice, and bring increased scrutiny from regulatory bodies who need to ensure safety and validity.

The recent dust-up between one of the few remaining genomics-based DTC companies and the Food and Drug Administration helps focus attention on what is at stake. Here we consider some early lessons emerging from their clash, and suggest an approach to providing DTC tests in a way that benefits all stakeholders, *i.e.*, DTC tests that would provide information of value in empowering individuals to better manage their health in the context of the current healthcare system in a manner that might be welcomed by those with regulatory concerns about safety and validity. 

The yet-unrealized potential for some form of more “personalized” DTC tests to fundamentally alter the practice and economics of healthcare is undeniable. However, it is also clear that there are serious issues—though perhaps not as many as feared—to be resolved if DTC testing is going to be successfully integrated into the current healthcare system.

## 2. Defining “Direct-to-consumer” Diagnostics Tests

Although obvious, it is important to note that DTC tests are, by definition, a set of tests requested *by patients* for their own decision-making. Tests promoted to patients but ordered by physicians are not strictly DTC tests, at least for the purposes of what we are considering here. In addition, a DTC test should be affordable and easily done (even multiple times, in some cases). It should also be accurate and actionable, even if the action suggested is as simple as a timely visit to a physician, who (it is hoped) has at his or her disposal enough biomedical knowledge to correlate the results of the test with appropriate disease management. We recognize that this may be a tall order in some cases, particularly as reflected in recent events related to certain genotype-based DTC testing.

## 3. DTC Lessons from 23andMe

At least 27 “DTC” companies currently offer, or have done so in the past, genetic testing either “directly” to consumers or via a doctor’s order [6,7]. Several of these, most notably 23andMe, have advertised their offerings in both high-profile traditional and social media, which has raised public awareness of the availability of genetic tests, though perhaps with less awareness of what the tests actually offer. Such advertising has also brought increased scrutiny from the U.S. Food and Drug Administration (FDA), which recently demanded (22 November 2013) that 23andMe stop selling its DTC health-related offerings until further validation studies are provided [8]. On 6 December 2013, 23andMe announced that it would continue to offer its test to consumers and provide both ancestry information and raw genetic data back to its clients, but would no longer offer health-related interpretation of data [9]. On its face, this action seems to partially satisfy the FDA demands, as agency representatives have stated that they believe individuals have a right to their own genomic data, but not to a testing firm’s unvalidated medical interpretation of those data [10]. However, before drawing any lessons from this event, it is perhaps worth revisiting what, exactly, the 23andMe DTC test is.

Initially, DTC genetics companies grew out of the research findings from genome-wide association studies (GWAS). In essence, the research-based association of specific polymorphisms in the individual’s genome with common diseases and traits formed the basis for several companies’ initial test offerings. Larger GWAS studies in multiple populations have continued to refine both the correlations of specific genetic variations with disease, as well as the scope of DTC tests offered. Companies providing these tests run the gamut of types of information offered, from ancestry to “nutrigenetic” evaluations [1,11]. Although the earlier offerings were largely dismissed by the medical community as “recreational genomics” [12], the continuing improvement in the quality and quantity of what is offered by at least some of them has forced medicine and health care to begin to confront the new challenges raised by their availability.

This growing awareness on the part of those who embrace genetics-based DTC tests and those who raise serious concerns about their value is further intensified by the astounding technological advances in genetic technology, particularly in whole genome or whole exome sequencing (WGS/WES). These advances have brought the cost of this higher level of genomic resolution closer to the cost of traditional GWAS studies. Although no company currently offers its WGS technology on a DTC basis, the FDA has recently approved the use of several of Illumina’s next-generation sequencing technologies as devices for the diagnosis of cystic fibrosis [13].

So where did 23andMe run afoul of the rules? Although 23andMe’s tests, like those of other companies currently or previously in this market space, are generally sold to consumers under the appealing idea of “empowerment” (e.g., “owning” your own genomic information—[7,14], it is not at all clear what consumers are actually empowered to do with the information they receive from such tests. Indeed, the whole notion of empowerment implies that what is delivered has sufficient value to drive decisions and actions. Granted, there is some evidence that ~80% of users feel some satisfaction or empowerment from genetic tests and the interpretation they receive [15]. Yet the actual utility of the information offered, at least in GWAS-based testing, also appears to be fairly low, both scientifically and in surveying the actions of recipients after they get their results [15,16,17].

One place this phenomenon is seen most clearly is in the subset of those customers who purchase such tests and then share the test information they receive back from the companies with their doctors. An interesting fictional “case study” published in the *New England Journal of Medicine* makes the point most succinctly: The best response that most doctors can give now to the question of what the majority of these genetic variations mean is “ask again in a few years” [18]. For the time being, at least, the real worth of these measurements is scientific: They are helping researchers dissect what genes (and variants) actually matter for human health [19]. As a diagnostic tool, there is simply still too much uncertainty around assigning real risk, especially in common diseases. Thus, the primary value of such studies is not primarily the prediction of individual risk but the discovery of biologic pathways underlying human disease. From these data we may ultimately derive good diagnostic tools, assuming that a way to incorporate environmental interactions in the analysis is developed (if environmental effects are as important as surmised [20]).

However, 23andMe appears to have overreached by offering health-related interpretations of the raw genomic data directly to consumers, crossing an uncertain boundary between information and true “diagnostics”. That, more than anything else, appears to have captured the attention of the FDA. In addition, as the FDA’s 22 November letter indicates, 23andMe inexplicably “went silent” for months on the FDA’s requests for further information and validation studies, leading Forbes’ Matt Herper to wonder if the company is “guilty of the single dumbest regulatory strategy I have seen in 13 years of covering the Food and Drug Administration” [21].

23andMe was building something of real value, we believe, analogous perhaps to the early days of basic human anatomy. They knew that to truly unravel the correlations of genomic variation with various disease processes they would need a massive database of genetic variation derived from potentially millions of people, with as much associated clinical data as possible. It would be a shame if those visionary efforts were stymied by a wrong-headed marketing strategy that proffered something to consumers that simply was not deliverable in these early days. The repercussions of the 23andMe/FDA dispute have yet to be measured, and yet we know that the health value of deep genetic analysis of human populations will be crucial.

## 4. Initial Thoughts on a Way forward with Protein-Based DTC Testing

On the positive side, the 23andMe-FDA interactions have helped us crystallize a bit more our ongoing thoughts about how one could set up a DTC strategy that could ultimately have a positive impact on individuals’ health ownership, the effectiveness of health care providers, and the important concerns of the regulators.

We should first note that we have spent the past 14 years focused on developing “phenotype-based” tests, in particular the measurement of protein changes in the body and the correlation of those changes with a variety of diseases and conditions [22,23]. We, along with others [24], believe that the “real time” measurement of health status as reflected in changes in protein levels will ultimately have more immediate clinical utility. We also believe that phenotypic DTC tools based on real-time measurements of protein changes in the individual would be more in line with “traditional” personalized medicine than gene-based offerings [25].

**Figure 1:**
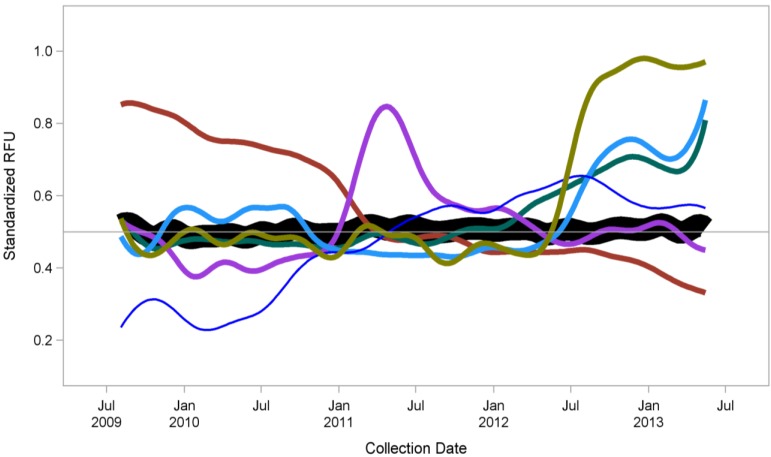
Proteomic profiling over four years (monthly sampling) from a single individual using SomaLogic’s SOMAmer^®^ reagent-based SOMAscan^™^ proteomic assay [23]. Regular measurements of 1,129 proteins revealed that most proteins remained stable over time (n = 900, black line). However, several smaller groups of proteins (colored lines) demonstrated changes, all of which except one group could be correlated with medical events (either before or after the specific associated event). A manuscript describing these data in more detail is in preparation.

Figure 1 shows just one example from our work: We have measured the blood based protein concentrations of a single individual regularly over a period of several years. As shown, the vast majority of the >1000 proteins we measured did not change (black line). However, there were groups of proteins that did change (colored lines), and the majority of these changes can be correlated with specific health-related events. Of note, these changes can be detected either after a particular event or, in some cases, preceding it (manuscript in preparation). This kind of longitudinal monitoring of real-time changes in an individual could lead to both early detection of disease onset, as well as monitoring real biological changes in response to diet, drugs, fitness changes, *etc.*

The difference between such phenotypic tests and ones based on assessing genotype is perhaps best summed up by Dr. James Evans at the University of North Carolina, in an interview with GeneWatch [26]:

*“…your doctor doesn’t check your cholesterol because they are primarily seeking predictive information. Your doctor checks your cholesterol because they can change your cholesterol. They aren’t doing it just so they can say, ‘Oh, you’re at increased risk for a heart attack. Have a good day.’ They are checking it because they can do something about it. And that puts it in an entirely different category than these direct-to-consumer genetic tests”*.

Although cholesterol may not be the best example, particularly in light of the new guidelines recently issued by the American College of Cardiology and American Heart Association [27], Dr. Evans makes an important point. The measurement of phenotype is a measure of the real time state of health of the individual being tested. From the humors of Hippocrates to the 3,000 or so molecular (often protein or metabolite concentrations) tests offered today by Quest Diagnostics [28], medicine has always relied primarily on measuring and interpreting accessible phenotype [29]. Indeed, the current focus on genomics as the primary delivery vehicle for personalized medicine [4] is a bit mystifying to us: Medicine has always been “personalized.” A good physician has always done a careful reading and interpretation of the phenotype of the patient in front of him/her, using the tools and knowledge available at the time. However, in the context of DTC offerings where a physician is not involved at the moment, there are new challenges to be addressed that are hinted at in the 23andMe/FDA story.

To highlight these, we offer here a “thought experiment” in how to structure a phenotype-based DTC test offering that could offer some initial utility, but would lead to ultimately to diagnostic tests of real clinical utility. Suppose a “home” test were available at a local drugstore, grocery store, movie theater, or by mail, which could, for some small cost (either to the patient, his/her employer, an enlightened insurance company, as a gift from family or friends, *etc.*), measure most of the protein levels in your blood, and provide that information back to you. In a few cases, those findings might be interesting, a few maybe even clinically relevant (after all, there are a lot of tests out there already that measure specific individual proteins, though not all are as clinically useful as initially believed, e.g., PSA testing [30]). You would not get an in-depth interpretation of those protein levels, though you might get some reference to relevant literature for any information that may be relevant from other published research efforts. However, of even greater value: let us say you take that test again in another 6 months or year, and find out the levels of about 50 proteins have changed fairly dramatically. Again, you may not get a direct interpretation, though the changes that are seen might prompt a strong suggestion that a doctor visit is in order. Indeed, one goal of such an approach would be to help a patient enter the health care system only when it might be necessary, where less uncertain diagnostics do the opposite [31].

Suppose further that as many people do this kind of testing, the data from many people with many different health conditions is combined to generate a knowledge database of what those changes in the measurements might mean in the context of health and disease. In this context, these data are analyzed, published, and provided back to you (and your physician). This kind of regular testing, research analysis, and knowledge gain would continue to grow, particularly if there were a large number of public and private collaborators supporting this effort and sharing (de-identified) data openly. In essence, the individual would not only be gaining greater insight into his or her own biological status, he or she would be participating in a new kind of clinical trial that could lead, eventually, to real diagnostic tests of value that would pass regulatory muster (such as home pregnancy testing, which relies on the accurate detection of human chorionic gonadotropin (hCG) in the urine, or the recently approved first home test for HIV [32], which measures the presence of anti-HIV antibodies in saliva). We surmise that an open, ongoing dialogue with regulatory agencies, providers, and potential participants to hone such an approach would be in the best interest of all parties, and could lead to real and robust phenotypic diagnostic tests. The key word in this thought experiment is “changes”—like a changing mole, changing weight, changing aches and pains, the idea is that molecular changes will eventually be used to construct correlations with health status.

We also believe that this kind of DTC approach may be what 23andMe initially intended, although based more on honing risk prediction than measuring real-time phenotype. However, the marketing gloss they provided to bring in the kinds of customer numbers they needed to gain enough data to build meaningful interpretations unfortunately put the cart a good way ahead of the horse.

Interestingly, several companies are gearing up to offer phenotype-based DTC clinical laboratory tests, either through pharmacy store chains (Theranos—[33]) or by selling in-home testing tools (QuickCheck Health—[34], Scanadu—[35]). Because they are generally offering diagnostic tests that are already in clinical use (albeit only available currently under a doctor’s orders), it will be interesting to see how the regulatory agencies respond.

## 5. Conclusions

The emergence of DTC testing reveals the dynamically shifting boundaries between health care providers, consumers, and regulators. The fact that gene-based diagnostics, by and large, were the first entrants into this new world highlights the challenges faced, largely because of the inherent uncertainty around assigning relative risk of developing various diseases to uncovered genetic variants. Furthermore, genomics introduced a new lexicon into medical practice, one that is still foreign to most physicians and other health care providers. Throughout history, doctors have spoken the language of phenotype, not genotype, largely because phenotype describes “what is” in real time. One’s genetic makeup is, indeed, “personalized”, but whether that personalization translates into medicine in useful and actionable ways remains to be proven. It certainly is not good enough now, at least for regulatory purposes, as demonstrated by the recent FDA action against 23andMe.

However, if phenotypes can be measured via proteomic analysis, perhaps even earlier than traditional symptoms appear, a DTC testing approach could help provide a deeper, integrated form of truly personalized medicine. Though there are many challenges still to be met, the recent advent of robust proteomic technologies [23] could help make this a reality within only a few years, if pursued intelligently, purposefully, and collaboratively with all the relevant stakeholders.
